# Supplementary material for: Patterns of Intron Gain and Loss in Fungi
Source: PLoS Biol. 2004 Nov 30;2(12):e422. doi: 10.1371/journal.pbio.0020422 (PMC532390; doi:10.1371/journal.pbio.0020422)
Supplement: Table S1 — Also available at http://genes.mit.edu/NielsenEtAl/. (4.3 MB ZIP). [file pbio.0020422.st001.zip › NielsenEtAl/html/522.html]

AN4857.1.NCU01997.1.MG02627.1.FG08709.1


```
 CLUSTAL W (1.82) Multiple Sequence Alignments - Introns Inserted


Sequence 1: MG02627.1	689 aa
Sequence 2: FG08709.1	655 aa
Sequence 3: NCU01997.1	675 aa
Sequence 4: AN4857.1	751 aa
Alignment Length: 768 aa
Number Identitical Residues: 321 aa
Alignment Score (without introns) 16346


MG02627.1 	--------------------------------------MFRLNIARLSSGAPNRRLLTAT
NCU01997.1	--------------------------------------MLRQSIIAASHCPNAP------
FG08709.1 	--------------------------------------MLRNAMLRPSARATMR------
AN4857.1  	MRFILSLLLLLPFTTAGFSPTCIRVVTALAARPNDLFNKFQREICDQGCQPTVPHWDLWT
          	     :     . :::. :.:.    :: :: ...  . ::  :   .  .        :

MG02627.1 	TARIPSCLSCASLARP-------------------TRRI~PTAILPSIRTLQTQHTPSDF
NCU01997.1	VAGITIPPTRAATRSL-------------------VRTP1PGP------------SSGPF
FG08709.1 	------CRPRALMAPP-------------------YRRL~Q--------------SGGPF
AN4857.1  	RNNTFVPAVRSLMQTLNAPKQEEALLKLGDDAADIIKRQ~CGPKLEGRDICADGHTLAAF
          	          :     .:.....:  . ...::.  :     .   .        : . *

MG02627.1 	RPAVPP-----------------SPSSLG~GPRKARTYTRSRKWARRLFIASAVLGTLYA
NCU01997.1	EPLTPP-----------------SPSKLG~APYPARSYTRTRRWSRRLLILSSLIGVAYL
FG08709.1 	QTMRPP-----------------SPEELG~APRRAKEYQRGRRWARKFLIISAVGGIVYL
AN4857.1  	GNCFKTNFLKAAIANLPTLLPMVSEPVCR1KPVPARTYRRTRKWLRRFFYLSLATGIVYV
          	     ..  .:: :. .:  .  *       *  *: * * *:* *:::  *   *  * 

MG02627.1 	IDSQVYASGMGRSLRTFGTGLLVALDYKINFRPDPL--PLIGSSGTIEDLHRRNAERLFE
NCU01997.1	TDKYVYASGLARSLRTFGVGLVVAADYKLNFRPEPLSIPWIGIPEGIPELHRRNAERLSD
FG08709.1 	GDRQIYASGFTRSLRTFGTGLLVAFDYKLNFRPQ----PITG--GTVQDLHNRNAERLFH
AN4857.1  	FDNQFYASSITRTARTFSLGVLVALDYKINFRPNP---PLAG---SIADVHARNAERLSD
          	 *  .***.: *: ***. *::** ***:****:.   *  *    : ::* ****** .

MG02627.1 	LLRKNGGLYLKIGQAIAMQSAVLPPEFQRMFARMFDDAPSVSWDEVERVIREDFGGRSVQ
NCU01997.1	LLRHNGGLYLKIGQAIAMQSAVLPPEFQAMFSRMFDDAPQDSWEEVEQVIREDFGGRSVE
FG08709.1 	VLRSNGGLYLKIGQAIAMQSAVLPPEFQKMFSRMFDDAPQDDWADIEAVIRQDFG-KSVE
AN4857.1  	LLRHNGGLYLKIGQAIAMQSAILPPEFQKMFARMFDDAPQNSWKDVEKVIREDFG-KSAE
          	:** *****************:****** **:*******. .* ::* ***:*** :*.:

MG02627.1 	DVFGVSFAGADGRGVMERSARASASVAQVHWARLSDGTEVAVKIQKPEIAKQIGWDLWSF
NCU01997.1	EVFGVSFSGAEGRGVMERTARASASVAQVHWARLPDGREVAIKIQKPEIERQIGWDLWAF
FG08709.1 	EVFGVSFIGKEGMGLMERKARASASVAQVHWARLPDGREVAIKIQKREIAKQISWDLWAF
AN4857.1  	EVFGVSFSGDPTKGVMERVARASASVAQVHWARLADGREVAIKIQKREIAQQIQWDLWAF
          	:****** *    *:*** ***************.** ***:**** ** :** ****:*

MG02627.1 	K2AVTWIYSWWFDLPFYSMVPFITERLELETDFINEAKNSDTMRDLIADEPYLRGRVYIP
NCU01997.1	K2VVTWVYTWWFDLPLYSLVPFISERLRLETDFENEAKNSEIMRQLVENEPSLRGRVYIP
FG08709.1 	K2TVAWIYSKWFDLPLYKMVPFITERLELETDFVNEAKNSEKMRELVNGEKSLRGRVYIP
AN4857.1  	K2VVTWIYSRIFDLPFYSLVPYVSERLFLETDFENEADNSEMMAKLVAGESRLRDRVYVP
          	* .*:*:*:  ****:*.:**:::*** ***** ***.**: * .*: .*  **.***:*

MG02627.1 	KVFSELSSKRVLVTEWIEGVRLWDREAMTRPWYGGYGKGSLGVNGTPLPSPDMAAVRAEL
NCU01997.1	PVYPELSSKRVLTTEWIEGIRLWDKDAMTRPWLDGYGKGSAGVHGAQLDPPDMPAIRREL
FG08709.1 	TVYPEFTTKRVLVTEWIEGIRLWDKKAMTSRWLGGHGNGSPGA-GSPLPKIDLEAARREL
AN4857.1  	KVWRELSSKRVMTAEWIEGVRLWDKDAITRTWRGGWREGSPGCGGTPLDSP------RAN
          	 *: *:::***:.:*****:****:.*:*  * .*  :** *  *: *            

MG02627.1 	RRNPE--SSKLKPERDEWRGRNGKGGLGLSKKDVMTTIVDLFSAQIFKFGVVHCDPHPGN
NCU01997.1	RKNTQGYTFMIKPERTEWKGRRGKGGLGLSTKDVMTTMVDLFSAQIFKWGVVHCDPHPGN
FG08709.1 	RTRPY--QENLKPERQEWRGRRGRGGLGLSSREVMTTMVDLFSAQIFKWGVVHCDPHPGN
AN4857.1  	RSAPARSSFKIKPERNDWRGRNGRGGLGLSLKEVMTTMVDLFSAQMFLWGLVHCDPHPGN
          	*  .      :**** :*:**.*:****** ::****:*******:* :*:*********

MG02627.1 	IFIRRLPGGRAELVLIDHGLYVYLTPKFRREYSEFWKALMTFDNKTIARVCDQWGIKGAD
NCU01997.1	VFIRRLPNGRAELVLIDHGLYVYMSDKFRHEYGEFWKALMTFDNNKIKEITDEWGIKAVD
FG08709.1 	IFIRRLPNGRAELVLIDHGLYVYMSQKFRNEYATFWKALMTFDNKTISRVTDAWGIKAAD
AN4857.1  	IFIRRKPNGHSELVLIDHGLYIHMDPEFRHQYARFWKALLTFDNATINEIVKGWGVNNPD
          	:**** *.*::**********:::  :**.:*. *****:**** .* .: . **::  *

MG02627.1 	LFASATLMRPYEGGD-GHYRRQLLGMDLPEDMTPKQRRFELQKRSKQAVREMLADENKWP
NCU01997.1	LFASATLLRPYEGGDDGGFQAKLMKH-LDGNKTASERSYEMQARMKQGIRDVLADEDKWP
FG08709.1 	LFASATLLRPYEGGD-GRTRNGIMKG--LEGKTPAERHYAMQQRMKQGIREILADEEKWP
AN4857.1  	IFASATLMKPYRGGN-LSTQQGIERL---SKAERAQRHYDMQQASRKAVREILGDETKWP
          	:******::**.**:    :  :            :* : :*   ::.:*::*.** ***

MG02627.1 	KELIFIGRNMRIVQGNNQHMGSPVNRVKMMGEWASRSMFEDRDRPFRERVANMWRHALFK
NCU01997.1	KELIFIGRNMRIVQGNNQYLGSPVNRIKMMGEWASRSSFQDPNLPLGQRLVNYWHHFLFK
FG08709.1 	QELIFIGRNMRIVQGNNQYLGSPVNRVKMMGEWASRSLFEDPHLPLSQRFQNRWRHLIFK
AN4857.1  	QQLIFIGRNLRIVQGNNQFLGSPVNRIKITGLWASRALVESPDLRLTEKIRNYGRHLLFR
          	::*******:********.:******:*: * ****: .:. .  : ::. *  :* :*:

MG02627.1 	TVLTASDVVFYFFKLRQFLGLGGGMEDEVEARMRQMAGDFGVELQHDVFAG
NCU01997.1	CVLALTDVAFYFFRVRQLLHRGGGMEDELEARMRDVAKEFGVELQHNVFEG
FG08709.1 	TVMAASDIAFYMFKLRQWLGLGGGMEDEMEKRMKDVASDFGIELQHDVFDG
AN4857.1  	VVLFSSDLFFWFTKVRQFLHLGGGMEDDLEAQMQSVAKDMGVELNHSIFEG
          	 *:  :*: *:: ::** *  ******::* :*:.:* ::*:**:*.:* *
```
